# Tailoring communication practices to support effective delivery of telehealth in general practice

**DOI:** 10.1186/s12875-024-02441-1

**Published:** 2024-06-27

**Authors:** Sarah J. White, Amy D. Nguyen, Peter Roger, Tim Tse, John A. Cartmill, Sarah Hatem, Simon M. Willcock

**Affiliations:** 1 Centre for Social Impact, UNSW Sydney, 704, Level 7, Science Engineering Building (E8), Kensington, NSW 2052 Australia; 2 Macquarie Medical School, Faculty of Medicine, Health and Human Sciences, Macquarie University, Sydney, NSW Australia; 3 Centre for Health Systems and Safety Research, Australian Institute of Health Innovation, Macquarie University, Sydney, NSW Australia; 4 St Vincent’s Clinical Campus, UNSW Sydney, Darlinghurst, NSW Australia; 5 Department of Linguistics, Faculty of Medicine, Health and Human Sciences, Macquarie University, Sydney, NSW Australia; 6 Department of Primary Care, Faculty of Medicine, Health and Human Sciences, Macquarie University, Sydney, NSW Australia

**Keywords:** Primary care, Family practice, General practice, Telehealth, Telemedicine, Guidelines, Communication

## Abstract

**Background:**

The unprecedented increase in telehealth use due to COVID-19 has changed general practitioners’ (GP) and patients’ engagement in healthcare. There is limited specific advice for effective communication when using telehealth. Examining telehealth use in practice in conjunction with perspectives on telehealth as they relate to communication allows opportunities to produce evidence-based guidance for optimal use of telehealth, while also offering practitioners the opportunity to reflect on elements of their communicative practice common to both styles of consultation. The objective of this research was to develop evidence-based resources to support effective, person-centred communication when GPs and patients use telehealth. This included examination of interactional practices of recorded telehealth consultations, exploration of GP and patient perspectives relating to telehealth, and identifying priorities for guidance informed by these analyses as well as participant co-design.

**Methods:**

This study involved recording telehealth consultations (*n* = 42), conducting patient surveys (*n* = 153), and interviewing patients (*n* = 9) and GPs (*n* = 15). These were examined using interaction analytic methods, quantitative analysis, and thematic analyses, to create a robust, integrated picture of telehealth practice and perspectives. The process of research translation involved a co-design approach, engaging with providers, patients, and policy makers to facilitate development of evidence-based principles that focus on supporting effective communication when using telehealth.

**Results:**

Three key themes relating to communication in telehealth were identified across the different analyses. These were relationship building, conversational flow, and safety netting. The draft best practice principles drawn from these themes were modified based on co-design feedback into five Best Practice Principles for Communication between GPs and Patients using Telehealth.

**Conclusions:**

Effective communication is supported through relationship building and attention to conversational flow in telehealth consultations, which in turn allows for safety netting to occur. In telehealth, GPs and patients recognise that not being co-present changes the consultation and use both intuitive and strategic interactional adjustments to support their exchange. The mixed-method examination of experiences through both a detailed analysis of telehealth consultations in practice and comparative exploration of GP and patient perspectives enabled the identification of principles that can support effective communication when using telehealth. Co-design helped ensure these principles are ready for implementation into practice.

## Background

Telehealth^1^ has become a widely accepted method for delivering and consuming healthcare services, emerging as a vital component of healthcare delivery during the initial stages of COVID-19 in Australia. The transition from predominantly in-person visits to telehealth occurred quickly [1]. This was followed by a marked increase in research to explore challenges and advantages associated with telehealth and other digital health technologies [2–4]. While there is high acceptance of telehealth by both GPs and patients [5–7], the nuances of effective communication in this context have yet to be incorporated into telehealth guidelines. This is due in part to limited evidence on how such consultations occur in practice [8]. The correlation between effective, person-centered communication and safe, high-quality clinical care is well-established [9]. This connection is substantiated by measurable clinical outcomes [10, 11] and heightened satisfaction levels for both patients and healthcare providers [12, 13]. Unlike telehealth consultations, in-person consultations between doctors and patients are conducted multimodally; that is, people are relying on all aspects of spoken and embodied interaction to participate in the consultation. There continue to be limitations for telehealth use, such as the inability to conduct a physical examination [14] and the potential for exacerbations of communication challenges [5, 14], particularly for already marginalised groups [15, 16].

Telehealth, by its very nature, means that individuals are not physically present (co-present) in the same space. Even on video calls, the lack of co-presence can alter the experience of a consultation beyond the obvious challenge to physical examination [17, 18] to more subtle aspects of communication [19]. When co-present, doctors will routinely do “being present” in their consultations, to “*presence oneself* with another means that one is available to understand where each person can at times share in the ‘being’ of the other” [20 p. 440, italics added]. In telehealth, this necessitates strategic adjustments in the communication to mitigate the risks associated with a lack of physical co-presence, as there is greater reliance on spoken words alone to signal the patient’s and doctor’s engagement with each other on a moment-by-moment basis.

A recent review of analyses of telehealth consultations in practice identified three key topics in such studies: “how participants manage (i) the interactional organisation, (ii) the therapeutic relationship, and (iii) the clinical activities.” (8 p.75).^2^ These findings are also mirrored in other observational and perspectives-based literature on telehealth﻿. Writing in the pre-COVID era, Sabesan et al. [21 p.101] noted that “the issue of doctor patient communication in technology is a complex and evolving topic” and recommended that future qualitative research explore the perspectives of both patients and doctors. They advocated reassuring patients that the telehealth consultation would cover everything that would be covered in a traditional consultation (i.e. the level of service would be the same). However, the impossibility of carrying out physical examinations and the fact that many telehealth consultations have no video component call such assertions into question. While many of the principles of doctor-patient communication apply equally to telehealth contexts [21], Duane et al. [22] noted that telehealth consultations take place in hybrid care environments that fuse elements of digital and physical space. As they point out, this means that many elements of non-verbal communication (hand gestures, micro-expressions, and gaze patterns, for instance) are attenuated, distorted, or lost altogether. It would thus be naïve to expect that communication dynamics in telehealth consultations will simply mirror those of traditional in-person encounters [18].

Telehealth use has increased substantially during and after the COVID-19 pandemic.^3^ A study using GP claims data in regional Victoria, Australia, showed telehealth use starting at 0% before the pandemic and peaking at 55% during the pandemic, with 25% of GP consultations during the 2-year study period during the pandemic being conducted via telehealth [23]. Qualitative research has been used to demonstrate patient and clinician perspectives of using telehealth during and after the acute phase of the COVID-19 pandemic across various specialties, highlighting barriers and facilitators to its ongoing use. There are numerous barriers, including, but not limited to, the lack of clear legal and regulatory frameworks [14, 24, 25], funding and reimbursement challenges [26], variable digital literacy [27, 28], and lack of access to technology [27]. Facilitators primarily centre around convenience and flexibility, such as reduced travel, cost-savings, increased safety (e.g. avoiding infectious diseases), comfort, and increased accessibility to care [7, 29–33]. Patients’ educational levels, type of health condition, and prior experience with telehealth also affected their experiences with this mode of healthcare delivery [34]. However, very little literature exists describing how the use of telehealth impacts the communication of participants [8].

Our objective was to develop evidence-based resources to support effective, person-centred communication when GPs and patients use telehealth. We used a mixed-method approach to create a comprehensive picture of the experiences of telehealth in general practice through: (1) identification of observable adjustments made to communication practices; and (2) exploration of GP and patient perspectives relating to communication. Through this, we identified aligning and contradicting aspects relating to perceptions of experience and observations of practice. These findings allowed for a robust understanding of what goes on as well as what matters for GPs and patients when communicating via telehealth to support development of co-designed principles to inform nuanced policy decisions and training activities.

## Methods

The study design was intended to capture multiple viewpoints of communication in telehealth. Unlike previous studies that focused on either perspectives or practice, our approach combines both aspects in a single, mixed-method study, employing multiple methods and types of data. Data included recorded telehealth consultations, patient surveys, and qualitative interviews with patients and GPs. The focus of this research was on metropolitan users, with rural and remote users of telehealth experiencing different facilitators and barriers [35] and with different pre-COVID-19 telehealth experiences [31], and thus necessitating separate examination. All data were collected within a metropolitan area in Australia with participants who were fluent English^4^ speakers and over the age of 18. These were examined using interaction analytic methods, quantitative analysis, and thematic analyses, respectively, with thematic synthesis of these findings. We took an appreciative inquiry approach [36] that “focuses on the positive attributes inherent within existing systems retaining and building on the facilitators or best elements of current practice” [37 p.2]. The analyses focused on how telehealth impacts practice of and perspectives on communication in GP consultations and how these findings can support effective communication in these contexts.

### Recorded telehealth consultations

GPs who regularly use telehealth for their consultations (> 2 consultations per week) were recruited via a direct approach within two metropolitan general practice clinics. The participating clinics were recruited with broad selection criteria of accredited general practices in metropolitan Sydney that include regular use of telehealth. Both clinics use electronic medical records that make available relevant clinical data for use within the telehealth consultations. Six GPs consented to participate. Each GP was asked to record (audio and video, if applicable) between 5 and 15 telehealth consultations. Patients who had booked telehealth appointments with the participating GPs were approached by administrative staff and asked to participate in the study prior to their appointments. Forty-two patients consented to participate, with 41 recordings having sufficient audio quality for analysis. These were recorded between December 2021 and May 2022. Thirty-five of these consultations were conducted over the telephone and thus were audio-only. This is reflective of the use of telephone versus video calls in general practice [38]. These were transcribed in strict verbatim format through a professional transcription service (Pacific Transcriptions) with excerpts for conversation analysis transcribed in Jeffersonian format by a member of the research team (SJW) [39].

In analysing the interactions, two approaches were used: interactional sociolinguistic discourse analysis (ISDA) [40, 41] and conversation analysis (CA) [42]. This multi-method approach focused on multiple levels of granularity of communication. The process involved all members of the research team independently examining numerous consultations using an inductive approach to identify aspects of interest in the consultation. The research team then participated in a data session identifying avenues to refine and pursue further analysis. This involved two investigators with training in ISDA and CA (PR and SJW) separately examining the data using each method to focus on the turn-by-turn sequential organisation of talk (in the case of CA) as well as the influences of the broader context and the alignments that participants adopted towards each other (in the case of ISDA) as the interaction unfolded. Such analyses often consider the action of the turns at talk; that is, we examine what the turn *does* in the unfolding interaction and *how* the turn achieves that action through aspects of turn design, sequential organisation, etc. When considering action, turns can achieve more than one action [43] and can be in the service of more than one agenda. In medical consultations we can identify clinical, relational, and interactional agendas [44], with the latter focused on how participants orient to the management of the ongoing interaction. The findings from these sessions were shared across iterative data meetings with the research team.

### Telehealth experience survey

A general patient survey asked patients to reflect on their most recent telehealth consultation.^5^ Two validated surveys, the CARE Patient Feedback Measure [45] and the Telehealth Usability Questionnaire (TUQ) [46], were administered together using an online survey platform (limesurvey) along with additional demographic questions and a final question for open comments. For the CARE survey, answers were scored on a 5-point scale (Poor to Excellent). A Likert Scale from 1 to 7 (Strongly Disagree to Strongly Agree) was used for the TUQ. The survey was distributed to 1975 patients from one of the participating GP clinics who had attended a telehealth consultation within the 12 months prior. Given participants were recruited from the same patient population, rates of telephone versus video call were likely similar rate of use more broadly [38]. We received 153 full responses, giving a 7.75% response rate. Descriptive statistics were used to analyse the numerical results (TT). There were 54 responses that included comments, which were analysed thematically by two members of the research team (SJW and SH).

### Qualitative interviews

Two members of the research team (AN and SH) conducted over-the-phone, semi-structured interviews with GPs and patients who had used telehealth in the 12 months prior. An interview guide was developed by the research team based on published literature and their own previous experience in telehealth research. Participants were invited to describe their experience of telehealth, which encompassed telephone consultations and those conducted via videoconferencing. Interview topics of discussion with GPs and patients included preparation for (i.e. organising of relevant information, training for telehealth, confirming correct person on the call); delivery of (i.e. delivery/receiving of information, difficulties faced, multi-tasking, topics of discussion), and ending telehealth consults (i.e. checking understanding, follow-up post-call, next steps). Participant recruitment involved contacting GPs through professional networks and contacting patient survey respondents who had nominated to be contacted for a subsequent interview. Patients who indicated interest in the interview were contacted by researchers. Data was collected across one of the two participating general practice clinics between February and November 2022. All telephone interviews were audio recorded and transcribed through a professional transcription service (Pacific Transcriptions) using intelligent verbatim. These transcripts were thoroughly and independently reviewed by two researchers (AN and SH) using thematic analysis [47] in NVivo 12, whereby each interview transcript was examined to identify initial codes. Reviewers then discussed these codes and categorised them into themes and subthemes through consensus, to form an analysis framework. All transcripts were then re-analysed using this analysis framework to ensure codes were appropriately included in the themes and subthemes. Any discrepancies were resolved by discussion. Interviews were conducted until thematic saturation, the point at which no new themes were raised by participants, was reached.

### Co-design of principles

Overarching themes were inductively identified through thematic analysis of the results from the different methods described above. This involved comparing and contrasting the results with a focus on aspects relating to communication in telehealth. To facilitate comparative analysis with numerical results of the survey, we thematically categorised the questions from the two surveys to align with the key themes identified across the other components, including the thematic analysis of patient comments in the survey. The analysis included examination of similarities and differences between stakeholder perspectives and observed practice.

The research team then transformed the three key themes and their associated findings into draft evidence-based principles for communication when using telehealth. These draft principles were presented at three stakeholder workshops for feedback, with two held via Zoom and one in person. Participants included healthcare providers, patients, policy makers, and medical students, with stakeholder types identified through an informal stakeholder mapping process. Each workshop consisted of a mix of different stakeholder types as participants. During each workshop, members of the research team provided a brief presentation of research findings and facilitated a discussion about the draft principles.

Following the workshops, researcher notes as well as workshop recordings and their transcripts were analysed with specific focus on identifying comments relevant to the following: aspects in the draft principles that participants thought were important and why; aspects not captured in the draft principles that the participants thought should be considered / included and why; aspects in the draft principles that participants considered unimportant and why; aspects in the draft principles that participants considered incorrect or needing clarification and why; and specific comments on wording / phrasing of the draft principles. The feedback identified through this analysis was then used to refine the principles.

## Results

Here we present the synthesised analysis from the multiple methods used, with the relevant findings presented under each theme. The three inter-related common themes across the findings are *relationship building, conversational flow*, and *safety netting*. These are presented with the associated data type and related findings in Table 1. The table shows the results from each method, yet under each overarching theme, we present a more integrated explanation of results were possible (e.g. the sub theme of *relationship building that mirrors in-person visits* incorporates the findings of *relational moments* and *rapport building*). While there is overlap across the findings in terms of these themes, at times the results from each of the methods did not align within the theme; that is, perceptions and observations of telehealth experiences were at times contradictory. This is presented when relevant.

**Table 1:** Overarching themes and component findings

Overarching theme	Data	Related finding
Relationship building	Recorded consultations	Signalling presence
		Relational moments
	Survey	Doctor-patient relationship
	Interviews	Lack of visual cues
		Rapport building
		Adjusting / compensating
Conversational flow	Recorded consultations	Dealing with non-mutual realities
		Conversational rather than transactional
	Survey	Comparison to in-person visits
	Interviews	Impact of multi-tasking
		Proactive strategies
Safety netting	Recorded consultations	Repeats as understanding confirmation
		Future in-person appointments
	Survey	Suitability for the presenting problem
		Safety
		Access
	Interviews	Suitability for presenting problem
		Patient preference
		Variations in comfort and use

### Relationship building

Relationship building refers to interactional practices and techniques that are designed to and/or result in an improved relationship between patient and GP. Relationship building is important as it increases trust between patient and GP, can have therapeutic impacts, and reduces the risk of complaints [48, 49]. Relationship building has an important clinical role to play. As O’Grady demonstrates, a doctor’s displays of empathy can elicit crucial elements of a patient’s history that might otherwise remain undisclosed, with the potential to undermine clinical outcomes [50].

#### Lack of confidence due to lack of co-presence

The analysis of consultations revealed that many GP participants engaged in behaviours that contributed to relationship building, even though findings from the interviews suggested that they were less confident in how they were managing relationships in telehealth. In interviews, GPs expressed discomfort with telehealth consultations due to the lack of visual cues, even in video calls, which made it difficult for GPs to interpret a patient’s response to information they were receiving.

*“If it’s on a telephone, you can’t see their expressions and that can be really hard because you don’t know if they’re upset with the question or if they’re getting annoyed with your question… It is more to do with not being able to always read what the patient means because you can’t see them. You can’t make them feel at ease in the same ways you would if they were in front of you.” (GP #10)*.

#### Impact of telehealth on the relationship

GPs reported that they were concerned about the impact of telehealth on relationship building, but patients were generally not concerned.

*“Certainly, not always feeling that you know what emotion is actually being exhibited because you only have the one sense - your hearing - to actually be using in a consultation. Whereas normally there’s body language and there’s facial expressions… So yeah, that is still a big difficulty with telephone consultations.” (GP#10)*.

In the survey, patients reported that their most recent telehealth consultation with their GP was excellent in terms of relationship building, with GPs showing interest, care, and making patients feel at ease (Table 2). Overall, patients expressed positive views about telehealth due to its convenience and the good relationship they had with their GP.

**Table 2:** Patient survey responses relating to questions relevant to relationship building

	Median	Mode	Mean	Standard Deviation
Thinking about the last time you had a telehealth appointment, how good was the practitioner at… (5 Point – CARE survey)				
Making you feel at ease (introducing him/herself, explaining his/her position, being friendly and warm towards you, treating you with respect; not cold or abrupt)	**5**	**5**	**4.35**	**1.01**
Being interested in you as a whole person (asking/knowing relevant details about your life, your situation; not treating you as “just a number”)	**5**	**5**	**4.23**	**1.16**
Showing care and compassion (seeming genuinely concerned, connecting with you on a human level; not being indifferent or “detached”)	**5**	**5**	**4.23**	**1.14**
Being positive (having a positive approach and a positive attitude; being honest but not negative about your problems)	**5**	**5**	**4.31**	**1.08**
Thinking about telehealth more generally (7 Point – TUQ survey)				
I felt I was able to express myself effectively	**6**	**6**	**6.11**	**1.13**
I think the visits provided over the telehealth system are the same as in-person visits	**4**	**5**	**3.95**	**1.95**
I feel comfortable communicating with the clinician using the telehealth system	**6**	**7**	**5.97**	**1.23**

CARE survey answers were scored on a 5-point scale (Poor to Excellent). TUQ answers were scored on a Likert Scale from 1 to 7 (Strongly Disagree to Strongly Agree)

#### Adjusting for lack of co-presence to support relationship building

In interviews, GPs reported making strategic adjustments during telehealth consultations to compensate for the lack of co-presence. Such adjustments included GPs providing opportunities for patients to ask questions, GPs assessing patient understanding by asking them questions or “quizzing” patients, and using small talk.

*“Particularly if I’ve talked about maybe a result or something and then I just ask them - I just touch base with them to clarify that they’ve understood what I’ve said and then before I end the consultation I’ll ask them if they have any further questions. Then I often tell them don’t hesitate to contact the clinic if you want, if you have any further issues or you wanted anything further clarified.” (GP #2)*.

*“Because I’m not writing that [treatment plan] down for them in a consultation on video or telephone, I’ll often ask them to repeat it back to me. Okay, so can you just repeat to me what it is we’ve agreed on, just so I know that you’ve got it clear in your mind?” (GP #5)*.

Though this was reported, these practices did not appear to occur frequently within the recorded consultations. Other adjustments to account for lack of co-presence were both reported by GPs and were observed in consultations. These adjustments, as described in the following, signalled engagement with the interaction.

GPs reported using methods unique to telehealth to improve understanding over the phone such as the use of “screen sharing” and sending links to websites to telehealth patients to facilitate discussion of difficult to understand concepts over the phone.

*“Sometimes I will send them fact sheets and they’ve got diagrams and information. There’s a chat, so we can either email that to them, or the video conference actually has a chat function where you can actually send the patients links and things whilst you’re talking to them over the video consult.” (GP #2)*.

Such an approach, which was observed in several consultations, engages the patient in the current task within the consultation, demonstrating trust through facilitated access to knowledge, and thus working to build the relationship between GP and patient. Similarly, a GP might adjust in response to a lack of co-presence in a more subtle way. In Excerpt 1, for instance, the GP is searching through the patient’s records for details of previous endoscopy procedures. As this consultation does not involve video, the patient is unable to see what the GP is doing.

#### Excerpt 1 (MQ-TELE21-12)


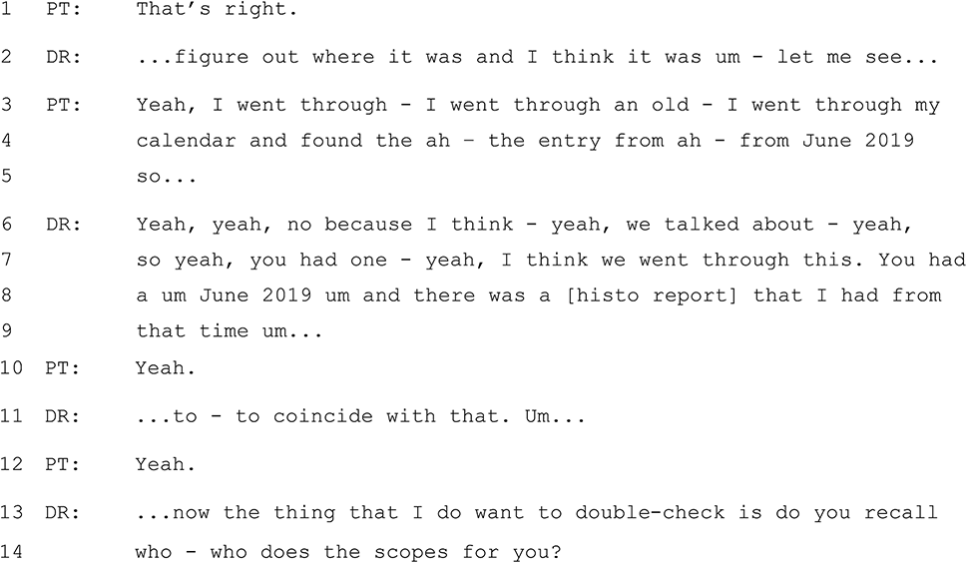


In Excerpt 1, it is notable that the GP continues to talk while searching through the patient’s record. In lines 6–9, the GP’s words are clearly not intended to provide information to the patient, but signal instead the GP’s progress in locating information from the files. In the telehealth context, this talk serves – on one level – simply to demonstrate to the patient that the GP is still ‘present’ in the absence of visual cues that would confirm this in an in-person consultation. A closer look at the GP’s words, however, indicates that they reference the shared history as part of the ongoing clinical relationship (e.g. in lines 6–7: “we talked about. … I think we went through this”). In lines 13–14, the GP chooses to engage directly with the patient in order to identify the individual who has previously done these endoscopic procedures, rather than continuing to search for this information on the computer. These moves on the part of the GP help to frame the current “referral” activity as building on the pre-existing clinical relationship, as evidenced by references to previous conversations and investigations.

#### Relationship building that mirrors in-person visits

Differences in relationship building techniques between consultations appear to be related to the relationship between the GP and the patient rather than telehealth vs. in-person. There were frequent references to the shared lifeworld (e.g., COVID-19 rules, restrictions, and protocols; daily life routines) – initiated by both GPs and patients. Patients felt that rapport was more important with their regular GP and less important with a one-time GP.

*“I didn’t find difficulty in raising more complicated issues with a long-standing relationship with the GP, that hasn’t been a particular difficulty at all I don’t think.” (Patient #2)*.

In the consultations we observed the use of “relational moments”. These are an interactional phenomenon first described by Gray et al. [51] with reference to communication in orthopaedic consultations. Medical students and doctors are often advised to engage in small talk with patients to help “build rapport” [52]. Yet, small talk occurs infrequently and, when it does, often serves not just the relational agenda, but also the clinical or interactional agenda as well [44]. Relational moments were observed across this dataset and can be defined as short sequences that reflect, maintain, and build the relationship between the GP and the patient.

Excerpt 2 occurs near the end of a consultation where the GP and patient have been discussing health behaviour changes to improve the patient’s health.

#### Excerpt 2 (MQ-TELE21-14)


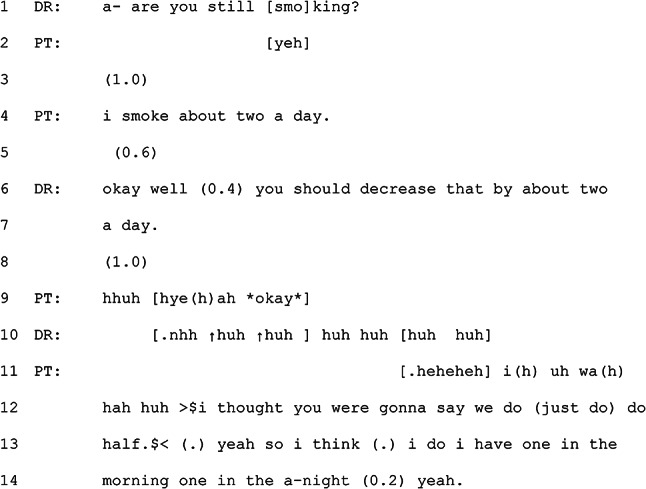


Rather than advising the patient on how to cut down smoking, the GP suggests that the patient should stop (line 6). This is done in a humorous manner as the GP starts the turn by suggesting a *decrease* which implies a reduction rather than a cessation but then completes their turn by making the reduction equal to the amount the patient currently smokes, essentially implying a cessation. The GP thus sets up a particular expectation in the first part of the turn and then subverts that expectation, creating what might be hearable as a joke. The patient responds to it as such, laughing after an initial delay (line 9). The GP joins in with laughing with the patient. The use of humour by the GP and its interpretation as such by the patient is a softer moment in a consultation where the GP provides their professional recommendation in a way that can be understood as humorous. This reflects the relationship between the GP and the patient, suggesting that the GP had a level of confidence that the joke would be received as intended. Relational moments such as this are not unique to telehealth, but their continued use within this different interactional environment demonstrates an orientation to the ongoing role of relationship building within these consultations.

### Conversational flow

Conversational flow refers to how the ongoing progress of the consultation is managed through interaction by patient and GP. Attending to conversational flow is important as it can support development of shared understanding and allows participants to manage interactional difficulties [19, 53]. Our analyses suggest that GPs and patients generally manage conversational flow well in telehealth, using strategies to mitigate the potential negative effects of phenomena such as talking over each other and multi-tasking, While interviews revealed that GPs were concerned about patient multi-tasking and its potential to distract the patient from the consultation, significant multi-tasking by patients were not observed in the consultations that were recorded, so we have insufficient evidence on how such multi-tasking might impact telehealth interactions. Patients felt that their interactions were significantly influenced by their location when they took the call and noted that they understood the necessity of maintaining focus and avoiding multi-tasking during consultations.

#### Managing conversational flow when multi-tasking

GPs reported multi-tasking during telehealth consultations, doing tasks for the consultation, such as typing up patient notes and looking up additional information. In interviews, GPs stated they took care to narrate what they were doing when they were multi-tasking to ensure patients knew the GP was still invested in the telehealth consultation.

*“I write notes at the same as I’m talking to people on the phone, so I would write notes at the same time. I think one of the good things about telehealth is that you can look stuff up simultaneously without interrupting the rapport, so yeah, I would often look up [guidelines] at the same time as I’ve got someone on the phone, or [medicines handbook] or look up [clinical resource] … You can still sort of listen while you’re looking up stuff.” (GP #4)*.

This was observed in the recorded consultations. In telehealth, as in all phone- and digitally-mediated communication, people conduct conversations within non-mutual realities [54]. That is, the participants do not have access to the environment from which the other participants are communicating. This includes the physical space, the activities going on around each participant or being conducted by each participant, the other people not on the call but who may be co-present, and technological difficulties or delays, any of which can impact the ongoing conversation. While this overlaps somewhat with the concept of *presencing*, the difference is in the integration with the relational component. That is, managing non-mutual realities is geared toward mitigating impact to the unfolding of the interaction rather than the ongoing relationship between the participants. When non-mutual realities impact conversational flow, the risk is that they are heard as doing something else or that the other participant has dropped off the call. This occurred numerous times within the data set and was primarily achieved by implicit or explicit referencing of what was occurring within the speaker’s environment that was impacting conversational flow. An example of this is shown in the following excerpt.

In the consultation from which Excerpt 3 is taken, the patient has presented with a request for iron level testing due to tiredness. The Excerpt begins just at the end of a sequence where the patient and GP have been discussing the current sleeping habits of the patient’s infant child. The GP then orients to the task of printing, without informing the patient what is being printed. Following the Excerpt, the discussion about the patient’s current work and schedule continues. The reason for printing is made apparent later in the consultation when the GP mentions that they have printed the blood test form for the patient.

#### Excerpt 3 (from MQ-TELE21-23)


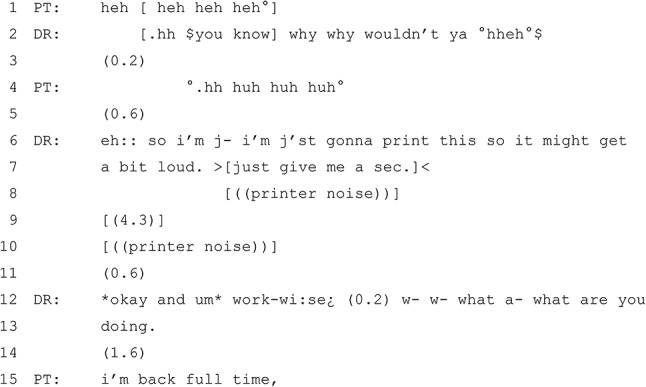


The GP and patient share mutual laughter about the patient’s child, who is frequently needing to be held by a parent in the evening. The GP is audibly typing throughout the previous talk.^7^ The GP then begins a new sequence that explicitly references the non-mutual reality of printing (lines 6–7). By foreshadowing the loudness of the printer and the impact it might have on the call, while also indicating that they need to pause the conversation (‘just give me a sec’), the GP manages the disruption of the conversational flow for the duration of the printer noise, with no talk occurring. This is followed by a 0.6 s pause before the GP moves to the next sequence.

#### Adjustments to account for being on the phone

In interviews, both patients and GPs reported that they managed conversational flow over the phone effectively, using various strategies to minimise or address potential issues. Such communication strategies included leaving space for each other to speak by providing long pauses, listening to each others’ tones, speaking slower and using a friendly voice.

*“Making sure that you pause long enough to let them speak, because you haven’t got those visual cues on the telephone, you have to often make a concerted effort to pause long enough to make sure they’ve said what they want to say, or if they want to add some more, and so it requires a bit more patience from that point of view, and I guess that’s why I prefer to only do that really with patients I know or know well because I’m familiar with their normal voice, and I can potentially pick up changes in the tone of their voice that would pick up on whether they’re concerned or whether they’re upset, because you’ve got to remember, yeah, you lose all those visual cues that you use to determine how a patient is feeling.” (GP #14)*.

Greater comfort with longer pauses is observed in the recorded data, which is reflective of this strategic adjustment to respond to potential issues in latency [19]. As can be seen in Excerpt 3, there is a longer pause (line 14) that is not problematised by the GP nor does the patient provide a ‘dispreferred’ response (i.e. one that does not align with structurally with the design of the previous turn [55]), which might be expected after such a pause [56].

The survey responses revealed that the patients did not find aspects of conversational flow, such as telling their story, problematic during telehealth consultations, even with occasional technical difficulties causing disruption (Table 3). Most patients considered telehealth as effective as in person consultations for communication and rapport, and they found it convenient. However, some patients felt the need for further engagement and self-advocacy during telehealth consultations.

**Table 3:** Survey responses relating to questions relevant to conversational flow

	Median	Mode	Mean	Standard Deviation
Thinking about the last time you had a telehealth appointment, how good was the practitioner at… (5 Point – CARE survey)				
Letting you tell your “story” (giving you time to fully describe your condition in your own words; not interrupting, rushing or diverting you)	**5**	**5**	**4.31**	**0.97**
Really listening (paying close attention to what you were saying; not looking at the notes or computer as you were talking)	**5**	**5**	**4.24**	**1.08**
Thinking about telehealth more generally (7 Point – TUQ survey)				
It was easy to learn to use this system	**7**	**7**	**6.29**	**1.10**
I could easily talk to the clinician using the telehealth system	**6**	**7**	**6.14**	**1.14**
I could hear the clinician clearly using the telehealth system	**6**	**7**	**6.20**	**1.17**

CARE survey answers were scored on a 5-point scale (Poor to Excellent). TUQ answers were scored on a Likert Scale from 1 to 7 (Strongly Disagree to Strongly Agree)

#### Conversationaility

Contrary to community concerns about telehealth being purely transactional, GPs and patients can still be opportunistic in their consultations, responding to different cues within the conversation to explore new topics. This reflects how GPs and patients maintain and adapt their skills as conversationalists to suit the modality of telehealth, retaining a sense of conversationality in their consultations. In our analysis, we identified segments of consultations that were not ‘transactional’ in that they involved talk that was not directly related to any apparent clinical goals. These segments contributed to the overall conversational flow of the encounter and allowed for exploration of other aspects of the patients’ lifeworld, including that which is clinically relevant. These can also be considered to play an important role in relationship building, as discussed below. Excerpt 4 is one such example.

#### Excerpt 4 (MQ-TELE21-12)


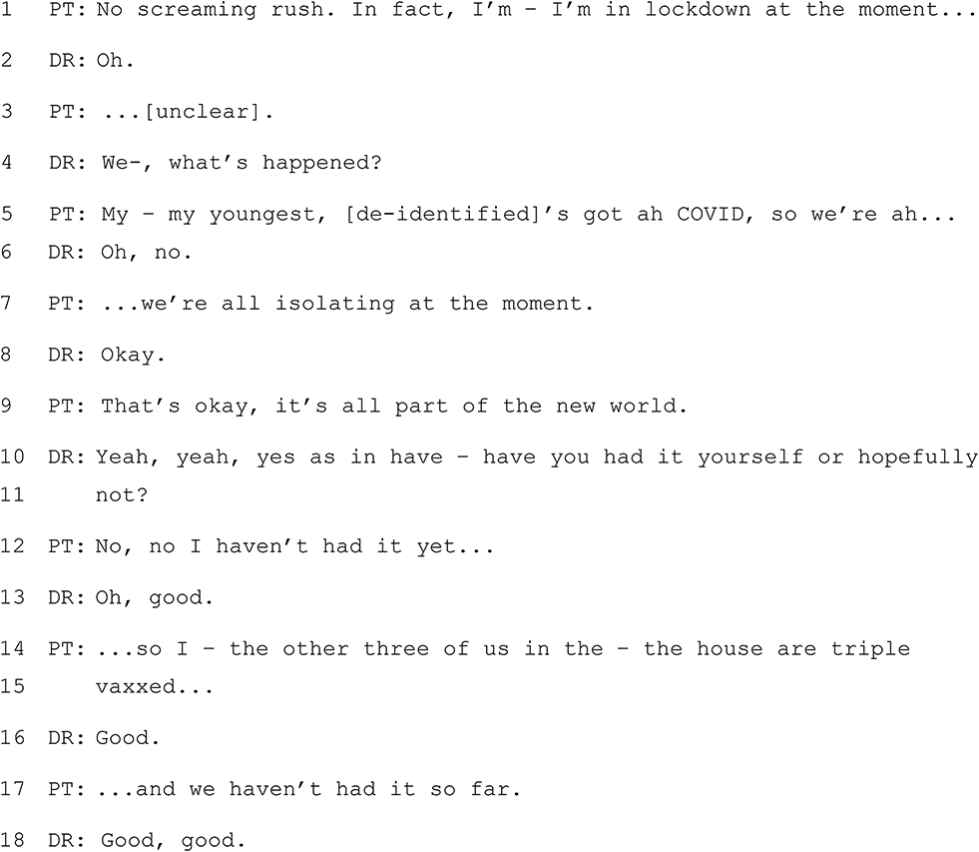


The interaction above occurs at the end of a consultation and follows discussion about how the patient will collect blood test request forms. The patient indicates that they are not in a hurry to receive the request forms, as they are not in a position to get the tests done immediately – the information about their child’s COVID and the family’s isolation is offered as an explanation for the lack of urgency. The frame is therefore one of information sharing, rather than advice seeking. It is interesting to note the reliance on shared “knowledge schemas” surrounding the public health advice and rules in place at the time (references to ‘we’re all isolating’ and being ‘triple vaxxed’ need no further elaboration). The GP in this case does not seek to shift the frame to one of “advice giving”, but they keep the conversation moving with: 1) requests for elaboration (e.g., ‘what’s happened?’ and ‘have you had it yourself, or hopefully not?’; 2) indications of paying attention without seeking to take the floor (e.g., ‘okay’); and, 3) affirmations (e.g., ‘good, good’). Importantly, the GP’s responses leave the patient with the option to invoke an advice seeking frame, but the patient does not choose this option (‘That’s okay, it’s all part of the new world’). There is also evidence of alignment between the two participants on the issue of vaccination.

### Safety netting

Safety netting involves ensuring patients and GPs have a shared understanding of the circumstances under which the patient needs to attend the GP in person, follow up over the phone, fill a back pocket prescription (i.e. a prescription provided for use if certain future criteria are met), or attend an emergency department. Safety netting is important as it supports the patient in accessing and receiving the care that they need in a timely manner [57, 58].

#### Suitability of conditions for telehealth

In interviews, GPs commented that only some conditions were suited to telehealth. There was variation, however, on what these conditions would be and on the level of comfort with and the use of safety netting. Individual patients and GPs favour the use of telehealth for some, but not all, health concerns, though the scope of which concerns are considered safe varied. Patients were concerned about problems that require physical examination while also noting that some consultations were easier to have via telehealth, which improved their healthcare experiences.

*“If I had an issue that I needed to go into lengthy discussion with the doctor, I would not do it on telehealth. If I had something that I had concerns about… I would much prefer to have a person-to-person discussion.” (Patient #9)*.

*“The consults I’ve had for telehealth were sufficient that they didn’t require a physical exam, so it was okay.” (Patient #3)*.

GPs and patients noted that telehealth was suitable for straightforward tasks such as for the purpose of getting a prescription or referral requests, and discussion of simple and routine conditions rather than complex acute issues.

*“If I was only needing a script or if I was only needing a referral… I would think that’s [telehealth] a very good way… All I need to say, it’s my time to see my specialist and my referral is out of date, could I please have another referral?” (Patient #9)*.

Patients and GPs also factored in patient personal preferences for telehealth or in person when deciding on the best mode of consultation. Such reasons for these preferences included convenience, reason for the consultation, travel distance to GP, and social factors.

*“I think that’s [simple indication] a very good time to have a teleconference because you’re not spending time going to the doctor yourself, and it’s very direct with the doctor then that’s the one thing I’m going to speak about and that’s it.” (Patient #9)*.

*“If you were going to have a meltdown, like be overwhelmed, it’s almost better to do it over the phone, because they’re not going to be able to react to you well anyway. Doctors can’t stay in high empathy alert for patients all day.” (Patient #1)*.

This was reflected in the survey responses, where patients were generally supportive of telehealth as a way to improve access and where patients reported that some conditions were not suited to telehealth. Patients gave slightly lower scores compared to other questions for feeling like the consultations were the same as those conducted in-person (Table 4). Some patients suggested the need for more information regarding which medical issues are best suited for in-person consultations and which are appropriate for telehealth.

**Table 4:** Survey responses relating to questions relevant to safety netting

	Median	Mode	Mean	SD
Thinking about the last time you had a telehealth appointment, how good was the practitioner at… (5 Point – CARE survey)				
Fully understanding your concerns (communicating that he/she had accurately understood your concerns and anxieties; not overlooking or dismissing anything)	**5**	**5**	**4.25**	**1.18**
Explaining things clearly (fully answering your questions; explaining clearly, giving you adequate information; not being vague)	**5**	**5**	**4.30**	**1.11**
Helping you to take control (exploring with you what you can do to improve your health yourself; encouraging rather than “lecturing” you)	**5**	**5**	**4.26**	**1.12**
Making a plan of action with you (discussing the options, involving you in decisions as much as you want to be involved; not ignoring your views)	**5**	**5**	**4.25**	**1.15**
Thinking about telehealth more generally (7 Point – TUQ survey)				
Telehealth improves my access to healthcare services	**7**	**7**	**6.13**	**1.29**
Telehealth provides for my healthcare needs	**6**	**7**	**5.60**	**1.51**
Telehealth is an acceptable way to receive healthcare services	**6**	**7**	**5.80**	**1.41**

CARE survey answers were scored on a 5-point scale (Poor to Excellent). TUQ answers were scored on a Likert Scale from 1 to 7 (Strongly Disagree to Strongly Agree)

#### Forms of safety netting

Safety netting took different forms, such as GPs triaging over the phone to determine suitability for telehealth or requiring an in-person consultation. The use of telehealth for specific health issues varied among individuals.

*“Well I guess the first part of the telemedicine consult is to kind of triage them and make that decision. So, it just depends on what the patient is presenting with. I mean classic examples are things like if a patient is presenting about a rash or something, well I can’t see the rash or feel the rash or examine the rash, so it’s going to be difficult for me to assess it over the phone. So then in that instance I’ll usually get them to come in. But the first part of the consult, the telehealth consult, is usually to determine whether the patient needs to come in to complete the consult, if they need some sort of physical examination, or if they’re in a grey area where you think they’re particularly unwell. Or is this something that can be sorted out over the phone, or over a videoconference.” (GP #11)*.

Safety netting in practice was observed in a variety of forms such as explicit requests for follow up, confirmation of key information, and opportunistic mentions of screening [59]. Explicit (on-record) acknowledgement of the limitations of telehealth was often observed, with reference to future in person consultations where tasks involving physical examination can be achieved. Excerpt 5 provides an illustration of how safety netting – in the form of explicit planning for a future in-person appointment – was introduced into the conversation by the GP (line 4) and elaborated upon in successive turns.

#### Excerpt 5 (MQ-TELE21-17)


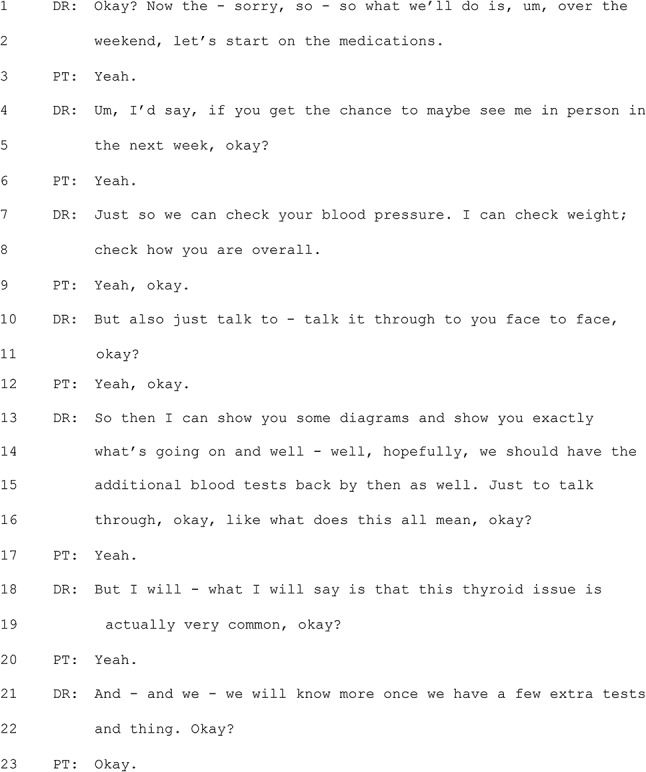


In Excerpt 5, the GP suggests the in-person appointment in line 4 and goes on to set out what they would hope to achieve at this future appointment. While some of the reasons involve monitoring of the patient’s physical condition (blood pressure, weight, and a general examination), the GP also flags the opportunity to engage in a thorough discussion (‘talk it through’ in line 10). The way in which the GP frames the future appointment suggests that safety netting can involve not only a concern for a patient’s ‘physical’ safety, but also the importance of the patient having as full an understanding as possible of their condition. Notably, the GP follows this up with a provisional reassurance: ‘this thyroid issue is actually very common’ (lines 18–19).

GPs and patients were also observed using the common interactional practice of repeats in a way that supported safety-netting of important clinical information. Repeats can be used to repair following misspeaking, mishearing, or misunderstanding, to agree or express affiliation, to confirm, to resist or assert epistemic authority, or to acknowledge a responsive turn and “prompt” more talk [60–63]. In the recorded telehealth consultations, repeats were used to demonstrate (or provide receipt of) understanding of key information, such as drug names and dates. This appears to be more frequent than during ordinary conversation [63] and may be used to mitigate the increased risk of mishearing in telehealth consultations, particularly as this technique was mostly used for specific details.

Excerpt 6 occurs in a consultation where the patient and GP discuss results from a gall bladder ultrasound and ongoing management of symptoms. This excerpt occurs near the start of the consultation as the GP asks for an update on how the patient has been feeling (physically) since their last appointment. The patient describes feeling unwell during that time and that the first medication of choice did not ease symptoms.

#### Excerpt 6 (MQ-TELE21-01)


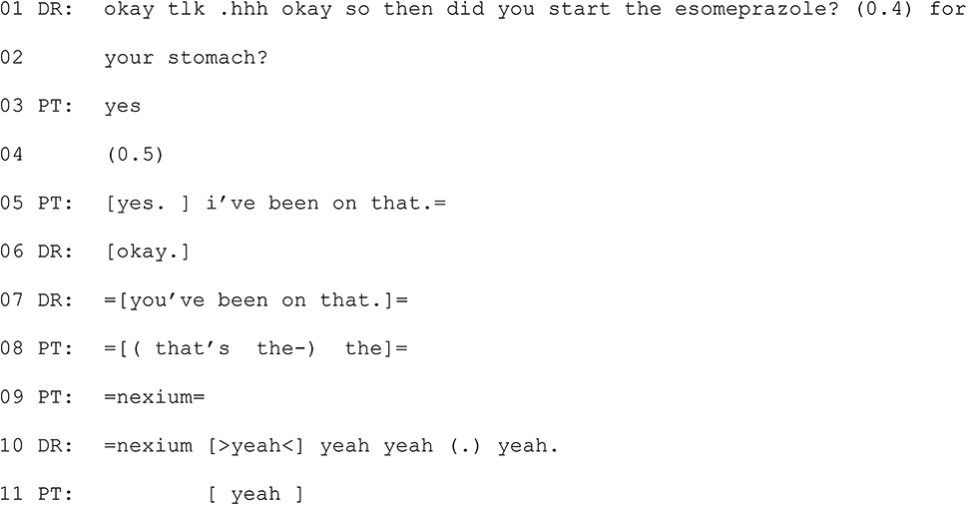


The GP asks the patient if they began taking a medication for treating reflux (line 1). The patient could have responded after the word ‘esomeprazole’ and the GP even leaves a space for the patient to do so (line 1). With a response absent, the GP pursues a response by referencing what the medication is for. The patient confirms that they have been (line 2), which implies that they understand the drug name used now that its purpose has been clarified. After another pause, the patient and GP both self-select as next speaker, overlapping each other. The patient provides additional confirmation, with ‘i’ve been on that’. This is repeated by the GP, but again in overlap, with the patient providing the brand name of the medication, ‘nexium’ (line 9). While this word is produced without overlap, it is preceded by overlapping talk, which introduces the risk of not being heard. In that local interactional context, ensuring that both the patient and GP are talking about the same thing is important for intersubjectivity (shared understanding) in the interaction as well as for clinical accuracy. Issues with latency in telehealth may play a part in increased orientation to the need to ensure certain turns have been accurately understood, with intersubjectivity being preferred over progressivity (continued progress), which is counter to the norm [64].

### Intersections between the three major themes

Conversational practices can also be used in a way that relates to more than one of the major themes, which is alluded to in the analysis of Excerpt 4 above. For example, safety netting can be a tool used near the end of a consultation to help move a consultation to closing, that is facilitating conversational flow, while also supporting a shared understanding of the next steps for the patient. Safety netting may also have a relationship building component with requests to follow up encouraging ongoing care. Similarly, relationship building can facilitate additional disclosure of patient concerns and increased trust between GP and patient, which also improves safety netting. Conversational flow can be supported by relationship building, with relational moments used to manage delicate interactional activities.

The intersections between relationship building, conversational flow, and safety netting can become a challenge if the GP and patient do not have a pre-existing relationship.

*“I’ve had a couple of telehealth [consultations] with different doctors that I’ve never met before and, you know, they seem to – they’ve obviously read my notes which is the important thing. You don’t really have time to build rapport as such.” (Patient #6)*.

*“The difference is when I’m talking to a patient that I actually know, particularly ones that I have a long relationship with, I can often understand the nuances of what they’re saying. Whereas with some of the patients that this – [de-identified doctor] used to take care of, I often have to really quiz them a little bit and you know ask for their patience in helping me understand exactly what do they mean about this or that or the other… If it’s on a telephone, you can’t see their expressions and that can be really hard because you don’t know if they’re upset with the question or if they’re getting annoyed with your question or anything – any of that emotion that can be behind some of the questions that we have to ask… You can’t make them feel at ease in the same ways you would if they were in front of you.” (GP #10)*.

These intersections are important to understand when considering advice for improved communication as conversational strategies are often doing more than one thing at a time, meaning provision of advice must reflect the possibility of intended and unintended consequences.

Similarly, other findings reflected previous work in demonstrating barriers and facilitators to telehealth, including difficulties using telehealth such as technological issues, financial considerations for GPs, concerns about clinical accuracy and continuity of care especially when the patient is new to the GP and physical examination is limited, and the need for support and training. These underpin the three major themes in that they indirectly impact access to and engagement with telehealth more broadly.

### Co-design and best practice principle development

Following the analyses and the identification of common key themes (see above, Table 1), the team drafted best practice principles to inform discussion within the planned stakeholder co-design workshops. These principles were designed to capture both the three themes and the specific findings that were used to identify them while also considering the intersections between them and other findings outside of the themes that underpin them (see above). The draft principles and related feedback are presented in Table 5.

**Table 5:** Draft principles and stakeholder feedback

Draft Principle	Summary of stakeholder feedback
1. Telehealth is best for consultations between GPs and patients with an existing relationship.	The participants thought this principle was quite appropriate and accurate.
2. Patients and GPs should avoid telehealth appointments if they do not know each other (well or at all).	Participants acknowledged that there is a higher potential of risk associated with telehealth consultations if the GP and patient do not know each other. It was suggested if telehealth is unavoidable, additional time should be allowed to establish the appointment parameters and to formalise relationship building and safety netting.
3. If a patient visits a GP in the same practice as their usual GP but does not know them, the GP should be clear that their notes will serve as clinical handover to their usual GP, and they should encourage the patient to book a follow up with that usual GP.	Participants supported this recommendation, recognising that it should be standard practice and is of increased relevance following a telehealth consultation where standard procedures such as physical examination will not have occurred.
4. Training and guidance for communication and relationship building in telehealth can highlight the similarities between in person and telehealth consultations.	Participants thought it was important to reassure doctors that this is a safe technology. They discussed the importance of having training and guidance around communication and promoting similar key messages to consumers.
5. Training and guidance for telehealth can reinforce the value of reflective practice regarding communication, with consideration of the impact of aspects of co-presence and non-mutual realities on a GP’s own interactional practices.	Participants discussed the idea that some people may not have English as their first language and this needs to be factored into determining the appropriateness of telehealth. It was also suggested that the wording of this principle should be simplified.
6. GP practices / professional bodies / government bodies can work toward creating guiding principles for consultations that are suitable for telehealth.	No comments.
7. Ongoing funding / support for telehealth should include consideration of remuneration for additional time spent on tasks when the patient and doctor have ended the telehealth interaction.	This was recognised as a very significant point.

Other issues raised by participants in the draft principle stakeholder co-design workshops more broadly included incorporation or consideration of: the best modality for any particular problem presentation; awareness of sensitive issues; increased consumer awareness of telehealth options available to them; increased community acceptance; factors relating to rurality; the national health literacy framework strategy; involvement of family members or carers that want to be part of the telehealth consultation; and, consumer informed consent and screen sharing as a visual way to help patients understand complex information.

The feedback was used to refine the principles, which are presented here with explanatory information for each principle.

### Best practice principles for communication between GPs and patients using telehealth

#### Telehealth is better suited for consultations between GPs and patients with an existing relationship

GPs can leverage existing relationships with patients to build additional trust and rapport within telehealth consultations. This is made possible through continued use of relationship building strategies and orientation to conversational flow. It implies existing mutual knowledge and trust and promotes appropriate safety netting. If telehealth is unavoidable between a patient and GP not previously (or well) known to each other, additional time may be needed to conduct the consultation and to establish parameters for the appointment. This can minimise the risk of unsatisfactory outcomes for the doctor and/or the patient. This “introductory” period would allow GPs to better understand the health literacy needs of the patient as well as their broader clinical and personal context, and essentially mirrors what is done when seeing a new patient in person. Given that telehealth restricts some of the observational input available to the doctor, such an introductory period is even more essential in a telehealth encounter with a new patient. If this patient will be returning to their usual GP for follow-up, the GP on the call should provide adequate notes designed to facilitate conversational flow and relationship building as well as continuity of care, with consideration of the challenges that telehealth brings, such as the lack of physical examination. If an electronic medical record is unavailable, consideration should be taken as to how to manage such information.

#### Training and guidance for communication and relationship building in telehealth can highlight the commonalities between in-person and telehealth consultations

GPs can be trained to make strategic adjustments during telehealth consultations to compensate for the lack of co-presence, such as providing opportunities for patients to ask questions and using relational moments. Patients are generally happy with telehealth consultations and interactional practices are mostly similar and/or manageable within current skill sets. Conversational flow techniques employed by GPs during telehealth consultations can be seen as extensions of what they normally do with in-person consultations. However, GPs may need to be more conscious in applying techniques to nurture and drive relationship building aspects over the phone.

#### Training and guidance for telehealth can reinforce the value of reflective practice on communication, with consideration of the impact of co-presence and non-mutual realities on a GP’s own interactional practices

Training can include strategies to manage conversational flow over the phone effectively, such as leaving space for each other to speak by providing long pauses, listening to tone, and managing non-mutual realities. This involves reflecting on how a GP demonstrates their presence and attentiveness in the absence of visual cues and how a shared understanding of presenting concern, diagnosis, and treatment is managed. This involves both *reflection-in-action* (the process of thinking and analysing while engaged in an activity or task) and *reflection-on-action* (the process of retrospective thinking and analyses on past experiences, actions, or decisions after they have taken place) [65]. While most interactional practices are very similar, awareness of and attendance to the impacts of the lack of co-presence on the telehealth consultation may improve aspects of relationship building and conversational flow.

#### General practitioners and patients each need to be active participants when implementing guidelines for each consultation when considering and using telehealth

The GP and patient should both actively participate in decision making to mutually determine what clinical presentations need an in-person consultation versus what can be managed via telehealth, including determining within a telehealth consultation whether an in-person consultation is more suitable. This includes supporting individual GPs to determine their own parameters for telehealth consultations. Each GP will have differing levels of comfort and confidence when using telehealth, and deciding between telephone and video calls, and should identify and communicate their preferences to patients. This is to ensure that GPs can effectively deliver care for all situations using telehealth, within their comfort zone.

#### Funding and support for telehealth should include consideration of remuneration for additional time spent on post consultation tasks following the telehealth interaction

Funding and support for telehealth should take into account the additional time GPs spend on tasks such as typing up patient notes and looking up additional information during telehealth consultations. Consideration should also be given to the financial implications of technological issues and the need for support and training in telehealth. Doctors reported that they frequently deferred tasks such as letter or report writing until after the direct interaction with the patient had ended, whereas if the patient were physically present this would have taken place while the patient remained in the consultation. Funding and workload models should reflect this.

## Discussion

We are living in an era where every sort of human-to-human communication is changing constantly through the evolution of digitally-mediated interaction and social media [66]. The adoption of telehealth is occurring against this much broader landscape of communication evolution that does and will continue to impact upon healthcare communication as well. For clinical consultations, which have primarily been conducted in person, the uniqueness of telehealth is that the participants are not in the same physical space and may not have any visual input. Thus, effective spoken communication is key to acceptable outcomes and the best practice principles presented above are designed to support this.

Telehealth involves use of a familiar modality, i.e. telephone or video calls, for many other types of conversations, and applies it to a clinical task that, for most GPs and patients, is usually conducted in person. We took a multi-methods approach to understand the different aspects that coalesce into the complexity of these interactions. The results shed light on the experiences and perspectives of GPs and patients using telehealth, highlighting both challenges and potential benefits of this healthcare modality. The in-depth analysis of recorded telehealth consultations demonstrated the ways in which GPs and patients work together using modified communication strategies to make telehealth work in practice. The interviews and surveys revealed that GPs and patients recognise the difference made through not being co-present and use both intuitive and strategic adjustments to communication to manage relationship-building, conversational flow, and safety netting.

Relationship building moves often align with the shared clinical goals of the consultation, in that they are offered in a way that moves the conversation forward. Telehealth (particularly without video) means that the GP needs to build relationships with patients in different ways. When consulting a GP, a patient will naturally seek a feeling that the clinician is present on a cognitive, affective, and behavioural level [67]. The analysis of consultations showed some adjustments to interactional practice are made by GPs and patients, such as presencing. For instance, a GP’s running commentary while searching for results during a telephone consultation served as a signal (in the absence of visual contact) that the doctor was cognitively present and engaged. In addition, frequent conversational references to lockdowns, COVID-19 precautions, isolation requirements, and vaccination recommendations in our data served to reinforce that both doctor and patient were co-present in a shared local environment, even though they were physically distant. For the most part, however, GPs and patients perceived differences in the relationship building as to be based more on the level of familiarity between the GP and the patient rather than on telehealth versus in-person consultations. This may be further explained through observations in the literature [22] that when patient and doctor meet in a shared digital environment (rather than in an office or clinic) the power dynamics become more equalised. This in turn may help patients to feel more empowered and willing to raise issues and self-disclose information, highlighting the already acknowledged need for empathy and a patient-centred approach to foster a therapeutic relationship in telehealth interactions [68].

Attending to conversational flow can support development of shared understanding and allows participants to manage interactional difficulties [8]. Conversational flow was, in part, supported within consultations through reference to non-mutual realities. GPs, who often multi-task as part of their clinical work such as looking up results, reported referencing these activities. This behaviour was observed in the interaction analysis and was also discussed in interviews conducted with the GPs. The GPs’ approach to making their multi-tasking activities clear to patients reflects a conscious strategy to bridging the non-mutual realities that are inherent to telehealth consultations. GPs and patients also conducted consultations in a conversational rather than transactional manner, demonstrating a shared orientation to the purpose of medical consultations and how they are usually conducted.

The orientation to safety netting in telehealth emphasises concerns about telehealth being fit-for-purpose and the need to select the right conditions and relationships for telehealth consultations. Safety netting supports the patient in accessing and receiving the care that they need in a timely manner. In the recorded consultations, safety netting in practice occurred frequently and came in a variety of forms such as explicit requests for follow up, letting patients know when to seek additional care, and opportunistic mentions of screening. Micro-level practices, such as repeats as understanding receipts, were also used specifically to enable the shared understanding of clinical information. Safety netting practices were also mentioned by GPs in the interviews. GPs stated they ensured appropriate in person follow-up especially for consultations that were more difficult to perform over the phone e.g. requiring physical examination, or for more complex conditions, especially where communication of agreement was hard to gauge e.g. long pauses from the patient during mental health consultations could be interpreted as due to latency in the call or because of a problem in the patient’s agreement to a treatment plan. The importance of effective safety netting in telehealth consultations raised concerns about its suitability for certain conditions and the need to select the appropriate conditions and relationships for effective delivery of care. When telehealth is used, clear communication and adequate coordination of care is needed to ensure patients receive appropriate follow-up and access to in-person services as required [14, 69].

We have not identified published research to date that analyses both the conversational and linguistic features of telehealth consultations (as distinct from in-person consultations) and how experiences of telehealth are perceived by GPs and patients. While the themes and principles are not novel individually, this research not only contributes to cumulative evidence on telehealth, but also involves the articulation of principles produced through a co-design process and designed to relate specifically to communication in telehealth in general practice. When taken together, these three overarching themes facilitate engagement with the complexity of communication and the provision of care using telehealth. Building on previous research specifically into telehealth in Australian general practice [e.g. 7, 38, 70, 71, 72], these findings evidence both the desire of patients and GPs to maintain, and the interactional importance of maintaining, strong therapeutic relationships, managing conversational flow, and establishing clear safety netting protocols. The principles that were developed from these findings and iterated through the co-design process align with existing advice in this space (e.g. [8, 14, 26, 72]). As such, they are not entirely new concepts but are supported further by the findings in this study along with evidence from other studies. In a climate where primary care has reducing continuity [73, 74], and where there are increasing options to accessing general practice through telehealth [75], we argue that the explicit articulation of these three themes and of what might appear to be obvious principles, is increasingly important. Established doctor-patient relationships are important for all of the above reasons, with seemingly a wider range of concerns more easily covered when there is an existing relationship between the GP and the patient. We argue that this is because telehealth involves GPs giving patients more responsibility for communicating signs as well as symptoms and in decision making about when to follow-up and/or seek additional care or to raise additional concerns [68, 76–78]. It also involves patients giving GPs more trust in how a patient’s own understandings of their problems will be interpreted in the absence of physical examination. These differences are managed through communication strategies that support development and negotiation of trust while ensuring shared understanding. The status of the relationship between the GP and the patient may impact how comfortable both parties feel with these changes from in-person consultations. This aligns with previous observations in the literature [21] that a pre-existing clinical relationship can make it easier to manage potentially difficult situations when they arise in telehealth consultations.

The limitations of this study primarily lie within the selection criteria, which excluded those who lived rurally, were not fluent in English, and consultations with those under 18. The result is that recommendations drawn specifically from this analysis may not be as relevant for people within those populations, who may face additional barriers to telehealth use [15, 28, 35, 79]. Due to the aim of the study looking specifically at telehealth, results from all methods were not compared to practice and perspectives of GPs and patients regarding in-person consultations. While linked surveys and reflexive interviews [80] had originally been planned, these were unable to be conducted due to low response and participation rates. These methods would have added further integration to the analysis, providing additional insights into the connection between experience, as captured in the recorded consultations, and perspectives, as captured in linked surveys and reflexive interviews. As we look forward to how these findings might be applied, changes to funding may influence for what purposes telehealth is used and so the results should be considered with those implications in mind.

The principles developed within this study are designed to improve communication between GPs and patients to ensure effective delivery of care via telephone. Funding may appear as less directly relevant to communication in telehealth, however, as identified in the analysis, and building on previous research [2, 81–83], funding challenges can impact the sustainability of telehealth services and the ability of GPs to effectively communicate with patients. Training and guidance were seen as essential in educating GPs in transfer of communication skills from in person to telehealth consultations. Acknowledgment of the commonalities between the modalities, and consideration and reflection of a GP’s own interactional practices ensures that communication skills a GP has developed throughout their career, can be used to facilitate their interactions during telehealth consultations when they are providing care remotely. The co-design methodologies of the guidelines also demonstrate the power of involving intended end-users in the development of materials and resources being created for their use.

Implementation of the principles could be supported through an online training module aimed at enhancing communication skills in telehealth. We propose a reflective practice model, enabling doctors to self-identify areas for improvement by engaging with research evidence and relating it to their individual practice. Through such a module, learners could be provided with a window into what happens and why in telehealth while also being provided with information about how such evidence can help with active experimentation for improvement. Suitable evaluation tools include minute papers [84], for identification of what was learned, and a validated self-efficacy questionnaire [85], to identify areas where learners have improved and areas requiring further expansion. Incorporating evaluation supports individuals and organisations to make their own adjustments to how they wish to engage with such a module to ensure it is fit for purpose.

Successful telehealth consultations rely on building relationships and attending to the flow of conversations, both of which facilitate the practice of safety netting. GPs and patients acknowledge the unique challenges posed by remote interactions and adapt their interactional practices to support effective communication when using telehealth. This facilitates the provision of person-centered care. By approaching telehealth as a “normal consultation” – i.e., not a second-tier version of an in-person consultation – emphasis can be placed on engaging in reflective and evidence-based strategies for continued improvement. This involves being conscious of the limitations of the modality and applying intuitive and strategic adjustments to manage the differences.

## Conclusions

We aimed to develop evidence-based resources to support effective, person-centred communication when GPs and patients use telehealth. This was achieved through identification of three overarching themes from a mixed-method approach, enabling development of draft principles that were specific to the Australian metropolitan general practice context. These findings and co-designed principles captured the broader relationships between identified observable adjustments made to communication practices and the analysis of GP and patient perspectives.

While integrating specific skills may improve communication when using telehealth, this needs to be supported through personal reflection so that individual GPs identify their own areas for improvement across the variety of telehealth consultations they may have. Our findings suggest that an established GP-patient relationship is foundational to the provision of telehealth. We emphasise the need to consider the implications of situations where such relationships are not possible, such as in newer models of virtual primary care where such continuity may not be a component of the model [75, 86, 87].

## Data Availability

The datasets used and/or analysed during the current study are not available due to ethics restrictions.

## Footnotes

1 In the context of this study, “telehealth” is defined as consultations between patients and general practitioners that are conducted via phone or video call. This definition encompasses both modalities as they were both used throughout the research and is not limited to the reason for the call.

2 Although this review was published following the completion of the analysis of the first two phases of the project described here, there is overlap between these three topics and the three themes identified in our own work.

3 While COVID-19 might be understood as simply a catalyst for increased telehealth use, the data in this project were also collected during times where there were “waves” of increased infection rates, which impacted both the use of telehealth and the stress on participating GPs.

4 As Australia is home to many Englishes, we do not specify this as “Australian English”.

5 Initially this was designed as a post-visit survey of patients who had been recorded, however there were administrative difficulties that resulted in a low response rate.

7 It is unclear how audible it is to the patient as the recording was captured within the GP’s office while the call was on speakerphone, potentially making the typing more audible on the recording than it may have been for the patient.

## Abbreviations

GP: General Practice/Practitioner
ISDA: Interactional Sociolinguistic Discourse Analysis
CA: Conversation Analysis
